# Formulation of phage cocktails and evaluation of their interaction with antibiotics in inhibiting carbapenemase-producing *Klebsiella pneumoniae* in vitro in Kenya

**DOI:** 10.4102/ajlm.v11i1.1803

**Published:** 2022-07-18

**Authors:** Noutin F. Michodigni, Atunga Nyachieo, Juliah K. Akhwale, Gabriel Magoma, Abdoul-Salam Ouédraogo, Andrew N. Kimang’a

**Affiliations:** 1 Department of Molecular Biology and Biotechnology, Pan African University Institute for Basic Sciences Technology and Innovation (PAUSTI), Nairobi, Kenya; 2 Department of Reproductive Health and Biology, Institute of Primate Research (IPR), Nairobi, Kenya; 3 Department of Zoology, School of Biological Sciences, Jomo Kenyatta University of Agriculture and Technology (JKUAT), Nairobi, Kenya; 4 Department of Biochemistry, College of Health Sciences, Jomo Kenyatta University of Agriculture and Technology (JKUAT), Nairobi, Kenya; 5 Department of Medical Microbiology Laboratories, Souro-Sanou Teaching Hospital, Bobo-Dioulasso, Burkina Faso; 6 Department of Medical Microbiology, College of Health Sciences, Jomo Kenyatta University of Agriculture and Technology (JKUAT), Nairobi, Kenya

**Keywords:** phage cocktail, antibiotics, *Klebsiella pneumoniae*, carbapenemase, absorbances

## Abstract

**Background:**

The development of alternative control measures, such as phage therapy or adjunctive therapy, is urgently needed to manage the dissemination of carbapenemase-producing *Klebsiella pneumoniae.*

**Objective:**

This study aimed to evaluate the therapeutic potential of formulated phage cocktails and their interaction with select antibiotics in inhibiting the growth of carbapenemase-producing *K. pneumoniae* clinical isolate in vitro in Kenya.

**Methods:**

The study was conducted from February 2021 to October 2021 at the Institute of Primate Research, Nairobi, Kenya. Phage cocktails were formulated based on the morphology and biological properties of precipitated *Klebsiella* phages. The efficacy of individual bacteriophages and phage cocktails as well as their combination with antibiotics were determined for their inhibitory activity on carbapenemase-producing *K. pneumoniae* (KP20).

**Results:**

The precipitated bacteriophages were members of *Myoviridae, Siphoviridae and Podoviridae*. Regarding the evaluation of the phage cocktails, the absorbances at 600 nm of the bacterial culture treated with the two-phage cocktail (2φ MA) ranged from 0.173 to 0.246 at 16 h and 20 h whereas it peaked from 2.116 to 2.190 for the positive control. Moreover, the results of the adjunctive therapy showed that the optical density at 600 nm of the bacterial culture treated with 2φ MA was 0.186 at 24 h post-incubation time while it was 0.099 with the bacterial culture treated with imipenem in combination with 2φ MA.

**Conclusion:**

This study demonstrated that the two-phage cocktail in combination with imipenem was able to synergistically delay the increase in carbapenemase-producing *K. pneumoniae* growth in vitro.

## Introduction

The development of alternative control measures is urgently needed to manage the dissemination of carbapenemase-producing (CP) *Klebsiella pneumoniae* because of the decrease in the effectiveness of antibiotic drugs.^1,2^ Bacteriophage therapy has, therefore, been proposed as an alternative strategy in controlling multidrug-resistant (MDR) bacterial infections, including MDR *K. pneumoniae* clinical isolates.^3,4^ Some of the beneficial effects of bacteriophages over antibiotics include their abundance, host specificity and exponential replication.^5^ However, the narrowness of phage host range and the ever emergence of novel pathogen variants will at minimum represent some limitations for phage therapy.^6,7^ This challenge can be managed by formulating phage cocktails that contain different phages infecting one species or by combining phages with antibiotics, which may result in a broad spectrum of activity.^8^ The characterisation of therapeutic phages in terms of their biology and bactericidal activity is mandatory before any preclinical trials because the application of non-characterised lytic bacteriophages may cause undesirable virulence as an adaptive response to bacteriophage infection.^5,9^ In other words, the development of effective strategies, such as phage growth parameters, host range spectrum, the presence of lytic proteins, formulation of phage cocktail and phage synergistic interaction with antibiotic to enhance phage efficacy, is a key prerequisite for optimal treatment of MDR infections caused by *K. pneumoniae*. Several studies have reported the advantages and drawbacks of phage cocktails in treating MDR bacteria.^10,11^ Unfortunately, bacteriophages employed as cocktail or adjunctive therapy have not been well investigated in the African continent to effectively control MDR bacterial infections.^12^ This study aimed to evaluate the therapeutic potential of formulated phage cocktails and their interaction with select antibiotics in inhibiting the growth of CP *K. pneumonia* clinical isolate in vitro in Kenya.

## Methods

### Ethical considerations

This study was not human or animal research. However, clearance was issued by the Nairobi City Water & Sewerage Company Limited (NCWSC/HR/TRG.14/Vol.8/14/MMM/ak) for collection of waste water samples.

### Bacterial isolate collection, phage isolation and purification

The study was conducted from February 2021 to October 2021 in the Phage Biology Research Laboratory at the Institute of Primate Research, Nairobi, Kenya. The clinical isolate of CP *K. pneumoniae* (KP20) was obtained from the stock of the recently published data in Kenya.^13^ Bacteriophages were isolated from waste water samples of Dandora Estate Sewage Treatment Works (Ruai) and Rongai effluent, Nairobi, Kenya. They were purified from waste water samples through spot assay and double-layer plaque method as described, previously.^14,15^

### Determination of minimum inhibitory concentrations for KP20

The minimum inhibitory concentrations of a panel of antibiotics including carbapenems for KP20 were determined using VITEK 2 Systems version 9.2 and AST-XN05 (bar code 1481424403205844) card (BioMerieux, Inc., Hazelwood, Missouri, United States), according to the manufacturer’s instructions. The detailed procedure is described in online Supplementary Method 1.

### Bacteriophage propagation and precipitation

Phage lysate (≥ 10^8^ PFU/mL or 10^9^ PFU/mL) was propagated in large volume, concentrated and cleaned up in the presence of sodium chloride and polyethylene glycol as described elsewhere.^14,16^

### Morphological characterisation of precipitated bacteriophages

The morphological characterisation of the precipitated *Klebsiella* phages was conducted at the University of Leicester, United Kingdom. Negative staining with 1% (weight/volume) uranyl acetate on 3 mm carbon-coated copper grids of the precipitated *Klebsiella* phages was conducted at the University of Leicester and the bacteriophages were visualised with a transmission electron microscope (JEOL UK Ltd., Welwyn Garden City, United Kingdom).^17^ Bacteriophages were then classified according to their morphology.^18^

### Bacteriophage host range determination

A spot assay was conducted to determine the host range of precipitated bacteriophages and phage cocktails as described by Kutter.^19^ Bacterial strains tested in our study included reference strains and MDR bacterial strains reported by Michodigni et al.^13^ and described in online Supplementary Methods 2.

### One-step growth experiment

A one-step growth experiment for determination of latent period, and burst size of precipitated bacteriophages, was performed as described elsewhere, with some modifications.^20^ Exponential-growth-phase culture of CP *K. pneumoniae* (≈ 2.98 × 10^8^ CFU/mL) and phage precipitate (≈ 10^9^–10^10^ PFU/mL) were mixed with bacterial suspension to obtain a multiplicity of infection (MOI) of 0.1.^14^ The suspension was incubated at 37 °C in a shaking incubator for 10 min at 120 rotations per minute following with centrifugation. The pellet was then suspended in 7 mL of fresh tryptic soy broth and incubated at 37 °C in a shaking incubator at 125 rotations per minute. At 3-min intervals, from 0 min to 36 min, 500 µL of the suspension was taken and phage titres were estimated.^20,21^

### Formulation of phage cocktails and evaluation of their in vitro activity on carbapenemase-producing *Klebsiella pneumoniae*

Three *Klebsiella* phages, designated as CPRSA, CPRSB and ESBLA, were used for the formulation of two-phage cocktail (2φ MA) and three-phage cocktail (3φ MB). The two-phage cocktail comprised CPRSA and CPRSB while the three-phage cocktail consisted of CPRSA, CPRSB and ESBLA. Bacteriophage cocktails consisting of two and three tested phage lysates were formulated by combining in equal ratios of 1:1 and 1:1:1. The evaluation of the phage cocktails’ effectiveness was carried out based on the method described by Merabishvili with some modifications.^22^ Two-phage (2φ MA) and three-phage (3φ MB) cocktails were composed and their ability to inhibit the growth of KP20 at its exponential-growth-phase was evaluated. Subsequently, the efficacy of two individual bacteriophages, designated as *Klebsiella* phage CPRSA and CPRSB at MOI 1.0, 0.1 and 0.001, was also investigated. Only broth media (tryptic soy broth) and individual bacteriophages represented the negative controls. Bacterial culture with a final resulting concentration of ≈ 2.98 × 10^6^ CFU/mL served as positive controls. The OD_600_ of the host bacterium was measured every 4 h for 24 h, and finally at 48 h, to assess the inhibitory activity of bacterial growth by both individual phages and phage cocktails. The different absorbances obtained at 600 nm were compared to the ones of the positive control sample. The different concentrations of individual bacteriophages and phage cocktails are summarised in online Supplementary Table 1. The experiment was performed in duplicate.

**TABLE 1:** Minimum inhibitory concentrations of a panel of antibiotic and carbapenemase produced to KP20, Nairobi, Kenya, February 2021 – October 2021.

Antibiotic family	Antibiotic agent	MIC	Detected phenotypes	Detected genotypes‡‡
Beta-lactams	Ticarcillin/Clavulanic	≥ 128	Production of carbapenemase	blaTEM† and blaOXA‡ blaNDM-1§, blaIMP¶, blaOXA-48††
	Piperacillin	≥ 128		
	Cefuroxime	≥ 64		
	Cefuroxime Axetil	≥ 64		
	Cefixime	≥ 4		
	Ceftriaxone	≥ 64		
	Cefepime	≥ 64		
	Aztreonam	≥ 64		
	Meropenem	≥ 16		
	Imipenem	≥ 4		
Quinolones	Levofloxacin	≥ 8	Resistant	NA
	Moxifloxacin	≥ 8		
Tetracyclines	Minocycline	8	Intermediate	
	Tretracycline	4	Resistant	
	Tigecycline	1	Susceptible	NA
Phenicols	Chloramphenicol	≥ 64	Resistant	NA
Trimethoprim/Sulfonamides	Trimethoprim	≥ 64	Resistant	NA

NA, not applicable.

† , *beta*-lactamase gene,

‡ , Oxacillinase gene,

§ , New Delhi metallo-beta-lactamase-1 gene,

¶ , Imipenemasegene,

†† , Oxacillinase-48 gene,

‡‡ , Data reported by Michodigni et al. (2021)^13^.

### Evaluation of inhibitory activity of phage cocktails in combination with antibiotics on carbapenemase-producing *Klebsiella pneumoniae* in vitro

The inhibitory activity of the phage cocktail alone, and in combination with imipenem or tigecycline was assessed on KP20 at its optimal exponential growth (OD_600_ ≈ 0.6). The two antibiotics meropenem and tigecycline (Glentham Life Sciences Ltd. Wiltshire, United Kingdom) were tested in combination with 2φ MA and 3φ MB. The phage cocktails 2φ MA and 3φ MB with optimal concentrations (MOI 0.1 and 0.001) were used in this experiment in combination with the single and double of the minimum inhibitory concentrations of imipenem and tigecycline. Sterile broth media (tryptic soy broth), bacterial culture treated with antibiotics, and bacteriophage cocktails represented the negative controls. Bacterial culture with a final resulting concentration of ≈ 2.98 × 10^8^ CFU/mL served as a positive control sample. The growth of the host bacterium was monitored at 3-h intervals, between 0 h and 24 h, and at 48 h of incubation by measuring OD_600_. The optical densities obtained at 600 nm were compared to the ones of positive and negative control samples. The different concentrations of phage cocktails and antibiotics are indicated in online Supplementary Table 2. The experiment was performed in duplicate.

**TABLE 2:** Spectrum of activity of individual *Klebsiella* phages isolated from Ruai and Rongai in February 2021 (CPRSA, CPRSB and ESBLA) and phage cocktail (2φ MA and 3φ MB) on reference strains and different bacterial species.

*Klebsiella* phage	*MRSA* NCTC 12493	*S. aureus* NCTC 1312	*S. aureus* NCTC 6571	*E. coli* NCTC 13353	*E. coli* NCTC 12202	*E. coli* ATCC 25922	ATCC 27850 *P. aeruginosa*	NCTC 13438 *K. pneumoniae*	K2	K3	K13	K16	K20 (KP20)	K4	K15
CPRSA	NS	NS	NS	NS	NS	NS	NS	NS	NS	NS	S	NS	S	NS	NS
CPRSB	NS	NS	NS	NS	NS	NS	NS	NS	NS	NS	S	S	S	INT	NS
ESBLA	NS	NS	NS	NS	NS	NS	NS	NS	S	NS	NS	NS	NS	NS	NS
2φ MA†	NS	NS	NS	NS	NS	NS	NS	NS	NS	NS	S	INT	S	INT	NS
3φ MB‡	NS	NS	NS	NS	NS	NS	NS	NS	NS	NS	S	INT	S	INT	NS

NCTC, National Collection of Type Cultures, ATCC, American Type Culture Collection, NS, non-susceptible, S, susceptible, INT, intermediate.

† , Combination of two *Klebsiella* phages,

‡ , Combination of three *Klebsiella* phages.

### Data analysis

A two-tailed *t*-test was performed to determine the significance levels of phage cocktails in combination with select antibiotics using GraphPad Prism version 9.2.0. (GraphPad Software, San Diego, California, United States). A *p* < 0.05 was considered as significantly different.

## Results

### Minimum inhibitory concentrations of antibiotics and detected carbapenemase genes

The minimum inhibitory concentrations of imipenem and tigecycline to KP20 were 4 µg/mL and 1 µg/mL (Table 1). We previously reported the presence of extended-spectrum beta-lactamase (ESBL) genes (blaTEM and blaOXA) and carbapenemase genes (blaNDM-1, blaIMP).^13^ The ESBL and carbapenemase genes produced by KP20 are indicated in Table 1.

### Morphological characteristics of precipitated *Klebsiella* phages

**FIGURE 1:**
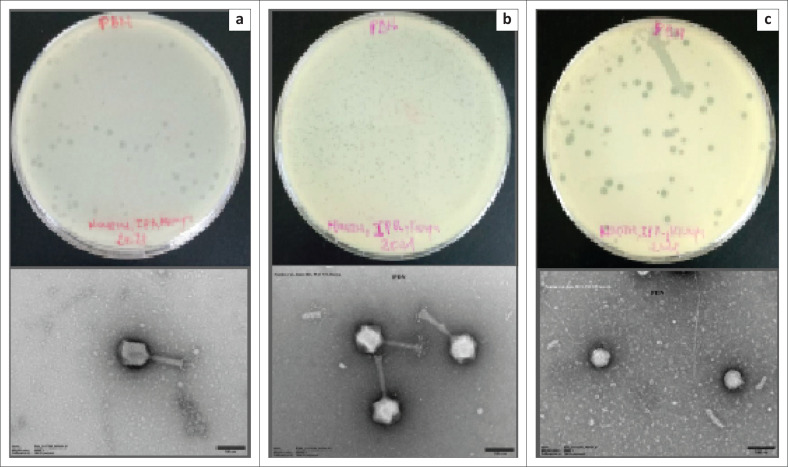
Transmission electron microscope images of the precipitated *Klebsiella* bacteriophages isolated from Ruai and Rongai in February 2021. (a) and (b) Bacteriophages belonging to the family of *Myoviridae* with contractile tails consisting of a sheath and a central tube (CPRSA and CPRSB); (c) and family of *Podoviridae* short tail (ESBLA).

A transmission electron microscope showed that the bacteriophages included in this cocktail were all tailed phages (Figure 1). Among the three imaged bacteriophages, two were myoviruses (CPRSA and CPRSB) and one was a podovirus (ESBLA).

### Precipitated *Klebsiella* phages growth characteristics

The burst sizes of the precipitated phages in the cocktail were 610 (1.812 × 10^11^ ÷ 2.98 × 10^8^), 1 (2.294 × 10^8^ ÷ 2.98 × 10^8^) and 10 (3.123 × 10^9^ ÷ 2.98 × 10^8^) for *Klebsiella* phage CPRSA, CPRSB, ESBLA. Regarding their latent period, *Klebsiella* phages CPRSA and CPRSB had the same latent period (9 min) and the one of ESBLA was 18 min as shown in online Supplementary Figure 1.

### Host range of *Klebsiella* phage lysates

*Klebsiella* phages CPRSA, CPRSB and ESBLA were used for the formulation of the two-phage cocktail (2φ MA) and the three-phage cocktail (3φ MB). The two-phage cocktail comprised CPRSA and CPRSB while the three-phage cocktail consisted of CPRSA, CPRSB and ESBLA. The two-phage cocktail was the combination of two bacteriophages belonging to the family *Myoviridae* while the three-phage cocktail was the combination of 2φ MA and one belonging to the family *Podoviridae.* The results of the host range analysis showed that both the individual phages and phage cocktails (2φ MA and 3φ MB) were active on bacterial strains belonging to *Klebsiella* species. The three carbapenem-resistant *K. pneumoniae* were susceptible to only one individual phage, the *Klebsiella* phage CPRSA. The two-phage preparations were also active on the three CP *K. pneumoniae* strains, in addition to K2, which was described as an extended-spectrum beta-lactamase producer in our previous study.

### Inhibitory activity of individual *Klebsiella* phages and phage cocktails on carbapenemase-producing *Klebsiella pneumoniae* culture

**FIGURE 2:**
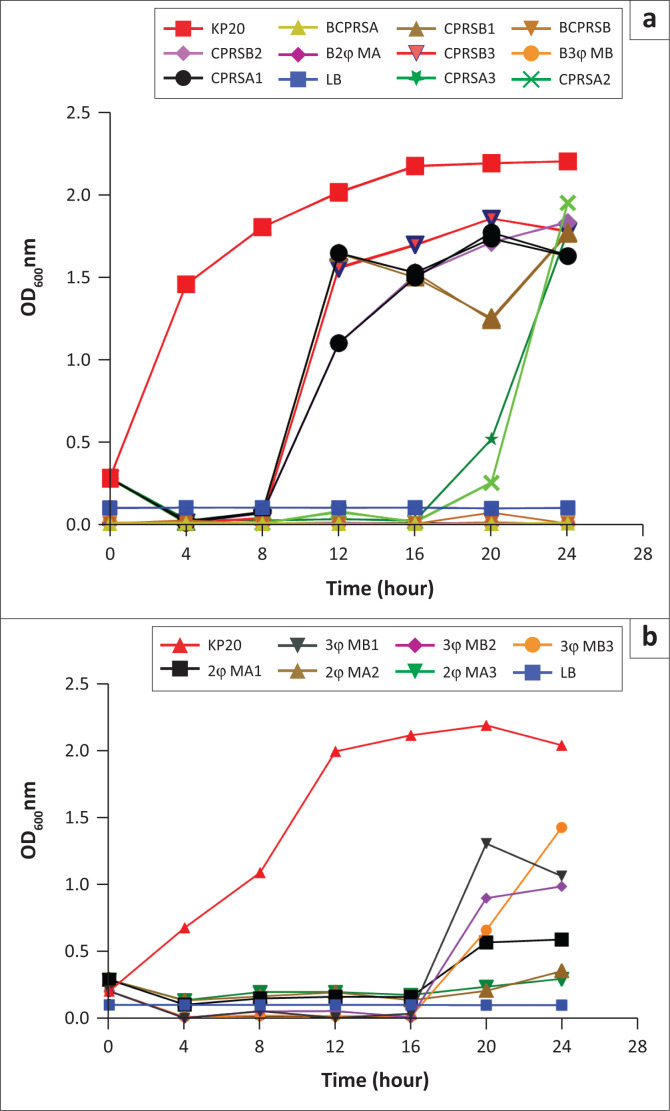
Efficacy of phage cocktails in inhibiting the growth of carbapenem-resistant *K. pneumoniae* (KP20), Nairobi, Kenya, February 2021 – October 2021. Optical density of KP20 cultures (≈ 10^6^ CFU/ml) infected with (a) Individual phages CPRSA and CPRSB at MOI 1.0, 0.1 and 0.001 (b) the 2-phage cocktail (2φ MA at MOI 1.0, 0.1 and 0.001) or 3-phage cocktail (3φ MB at MOI 1.0, 0.1 and 0.001). The digits 1, 2 and 3 coming next after the ID of the phages represents MOI 1.0, 0.1 and 0.001. The most effective phages were the individual phage CPRSA and phage cocktail 2φ MA at MOI 0.1 (CPRSA2 & 2φ MA2) and 0.001 (CPRSA3 & 2φ MA3).

The ability of the mixture of two phages (MA) and three phages (3φ MB) to inhibit the growth of KP20 culture at an absorbance of *≈* 0.3 was determined at three different multiplicities of infection (MOI 1.0, 0.1 and 0.001). It was observed in our study that there was an increase in CP *K. pneumoniae* growth to the individual phage CPRSB at its three different doses and to the phage CPRSA at MOIs 0.1 and 0.001 after 8 h and 16 h of incubation (Figure 2a). The two-phage combination (2φ MA) was able to control the rapid growth of CP *K. pneumoniae* after 16 h of incubation and up to 24 h (Figure 2b). This bactericidal activity of the phage cocktail was observed at MOIs 0.1 and 0.001 whereas the three-phage cocktail (3φ MB) could slightly control the increase in CP *K. pneumoniae* growth after 16 h and at MOIs 1.0 and 0.001. At MOIs 0.1 and 0.001, the dose response of the two-phage cocktail was not significantly different in the ability to inhibit the bacterial growth at this incubation time (*p* = 0.105). At the most effective phage dose (MOI: 0.001), the absorbances at 600 nm of the bacterial culture treated with the individual phage CPRSA, and the two-phage cocktail (2φ MA), increased from 0.019 to 0.515, and 0.173 to 0.246 at 16 h and 20 h, while it rose from 2.116 to 2.190 for the positive control during the same interval of time (Figure 2b). For the most active individual phage (CPRSA) and phage cocktail (2φ MA), the dose responses were not significant (*p* = 0.12) at MOI 0.1 after 16 h of treatment time (Table 3). Significant difference (*p* = 0.01) was observed between the two treatments at MOI 0.001.

**TABLE 3:** Statistical significance between the concentrations of individual phages (CPRSA and CPRSB) and phage cocktails (2φ MA and 3φ MB), Nairobi, Kenya, February 2021 – October 2021.

Treatment	*p*
CPRSA2† vs CPRSA3‡	0.11**
CPRSB2 vs CPRSB3	0.008*
2φ MA2 vs CPRSA2	0.01*
2φ MA3 vs CPRSA3	0.12**
2φ MA2 vs 2φ MA3	0.10**
3φ MB2 vs 3φ MB3	0.55**

* , Significantly different;

** , Not significant.

† , Digit 2 following the name of the phages represented MOI 0.1,

‡ , Digit 3 corresponded to MOI 0.0011.

### Interactive properties between the phage cocktails and antibiotics combinations in inhibiting the growth of carbapenemase-producing *Klebsiella pneumoniae* in vitro

**FIGURE 3:**
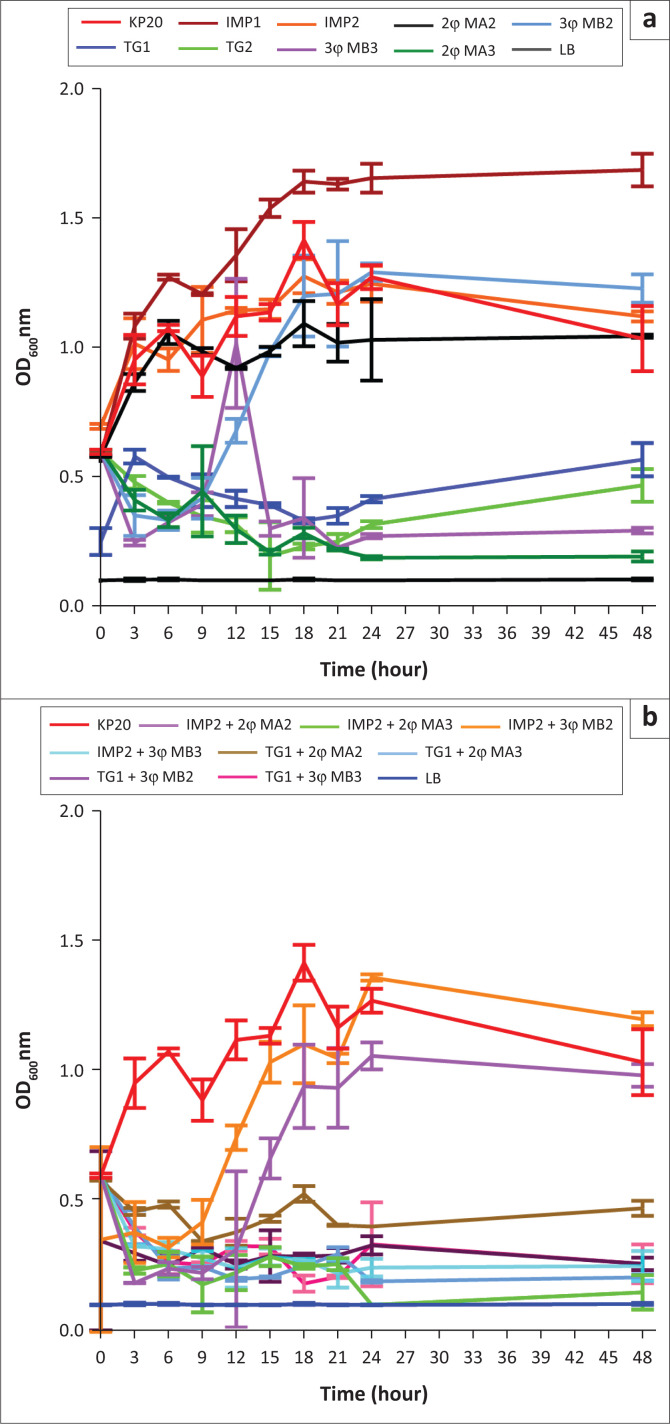
Bactericidal activity of *Klebsiella* phage cocktail alone or in combination with antibiotics, Nairobi, Kenya, February 2021 – October 2021. (a) Carbapenemase-producing *K. pneumoniae* culture (OD_600_≈0.6) treated with phage cocktail 2φ MA and 3φ MB at MOI 0.1 or 0.001, Imipenem (IMP, MIC = 4 µg/mL), and Tigecycline (TG, MIC = 1µg/mL), separately. IMP2 and TG2 were considered as double the volume of each antibiotic; (b) Carbapenemase-producing *K. pneumoniae* culture treated with phage cocktail + imipenem or phage cocktail + tigecycline. IMP2 + 2φ MA3 was the treatment of KP20 culture with the double volume of imipenem combined with two-phage cocktail at MOI 0.001, and TG1 + 3φ MB2 was the treatment of KP20 culture with the single volume of tigecycline combined with the three-phage cocktail at MOI 0.1.

The in vitro studies showed that the same phage cocktails (2φ MA and 3φ MB) maintained their bactericidal activity after 24 h of incubation at the lowest phage concentration (MOI 0.001) (Figure 3a). At different timepoints of 18 h and 24 h, the optical density at 600 nm of the bacterial culture treated with 2φ MA decreased from 0.217 to 0.186 whereas it increased from 0.226 to 0.269 for bacterial culture treated with 3φ MB. The results of the adjunctive treatment (phage and antibiotic combination therapy) experiments revealed that the absorbance of the bacterial culture treated with the combination of imipenem and two-phage cocktail (IMP2 + 2φ MA3) decreased from 0.256 (21 h) to 0.099 (24 h) at the lowest phage concentration (Figure 3b). When the phage cocktails were combined with tigecycline, the OD_600_ decreased from 0.294 (21 h) to 0.189 (24 h) for bacterial culture treated with tigecycline and the two-phage preparation at the lowest phage concentration, MOI 0001 (TG1 + 2φ MA3) while it increased slightly from 0.207 (21 h) to 0.331 (24 h) in the case of bacterial culture treated with tigecycline and the three-phage preparation (TG1 + 3φ MB3). The absorbances of the bacterial culture treated with tigecycline was 0.249 (21 h) and 0.314 (24 h) while it ranged from 1.212 (21 h) to 1.245 (24 h) for the bacterial culture treated with imipenem. The positive control sample exhibited an optical density of 1.166 (21 h) and 1.270 (24 h). The level of significance between the bacterial culture treated with 2φ MA and the bacterial culture treated with IMP2 + 2φ MA3 after 21 h of incubation are shown in Table 4. The results of bacterial cultures treated with phage cocktail alone, and in combination with imipenem, were statistically significant compared with the result of bacterial culture treated with only imipenem (*p* = 0.02). No statistical difference was observed between the results of the bacterial culture treated with a combination of phage and antibiotic and those of bacterial culture treated with tigecycline alone. A significant difference (*p* = 0.04) was observed between the bacterial culture treated with 2φ MA at MOI 0.001 and the adjunctive therapy (bacterial culture treated with IMP2 + 2φ MA3).

**TABLE 4:** Level of significance between bacterial culture treated with two-phage cocktail and the bacterial culture treated with two-phage cocktail and imipenem combination, Nairobi, Kenya, February 2021 – October 2021.

Treatment	*p*
IMP2† vs 2φ MA3‡	0.02*
IMP2 vs IMP2 + 2φ MA3	0.02*
2φ MA3 vs IMP2 + 2φ MA3	0.04*

* , Significantly different.

† , Digit 2 represented double of the volume of imipenem at MIC = 4µg/mL,

‡ , Digit 3 corresponded to MOI 0.001 of the two-phage cocktail.

## Discussion

This study aimed to evaluate the therapeutic potential of formulated phage cocktails and their interaction with select antibiotics in inhibiting the growth of the KP20 clinical isolate. On one hand, the phenotypic results revealed that the precipitated phages obtained in this study had lytic activity with high titers and they were related to tailed phages. The latter are generally divided into three families including *Myoviridae* with contractile tails consisting of a sheath and a central tube (25% of tailed phages), *Siphoviridae* with long and noncontractile tails (61%), and *Podoviridae* having short tails (14%).^23^ *Klebsiella* tailed phages identified in our study belonged to the family of *Myoviridae* and *Podoviridae*.^24^ Previous studies reported similar observations regarding the morphological characters of *Klebsiella* phages.^25^

On the other hand, this study showed that one of the precipitated phages (CPRSA) had the shortest latency period (9 min) and high burst size (610). Our findings were supported by Horváth et al. in 2020. The authors reported that their isolated *Klebsiella* phage had a relatively short latency period of 18 min and its burst size was ~220 phage particles per infected bacteria.^20^ Indeed, a high burst size is key to achieve a productive adsorption and replication of bacteriophages and to reach the benefits that phages could have in comparison with antibiotics while a short latency period is recommended from a phage therapy perspective.^26^

Both the individual bacteriophages and phage cocktails were not susceptible to the reference bacterial strains tested in this study and, hence, displayed high specificity for their host bacteria, which were KP20 clinical isolates. Previous studies on host ranges of *Klebsiella* phages reported similar results.^27,28,29^ The major consequence of phage host specificity is that it demands an appropriate diagnosis of the bacteria involved in the infection before initiation of phage treatment.^7,30^ Nevertheless, this narrowness of phage host range to the strains of the same bacterial species could limit its lytic activity on microbiota.^7,30^ The phage host range is indeed affected by a number of factors including the absence of required accessory proteins for phage adsorption, restriction-modification and clustered regularly interspaced short palindromic repeats (CRISPR) systems.^31,32^ Despite their narrow host range, bacteriophages with lytic activity may still be useful in phage therapy and the use of bacteriophages as cocktails for adjunctive treatment can represent a highly attractive strategy.^33^

Besides the choice of bacteriophages with different bacterial cell wall receptor recognition sites in formulating a phage cocktail, the most important criterion for successful phage cocktail preparation also includes the compatibility of bacteriophages in mixtures.^22^ Therefore, our study demonstrated that the mixture of two phages (2φ MA) was able to significantly delay the resurgence of bacterial cells in culture, as compared to the application of individual phage or the mixture of three phages (3φ MB) (*p* = 0.02). Our result was in line with the previous data published on phage cocktail efficacy.^11^ This synergistic activity of two-phage cocktail over the mixture of three phages in our study suggested that the individual phages might have employed different receptors to adsorb to the bacterial cells and, hence, effectively inhibited the bacterial growth. Some authors have reported that one phage in their two-phage cocktail used an outer membrane protein (OmpC) as a receptor, and another one employed a lipopolysaccharide component as its receptor to effectively control bacterial resistance.^34,35^ Moreover, the mixture of many phages may be less effective in the absence of identification of specific bacteriophage receptors because the same phages might share the same receptors and interfere with one another.^36,37^

The adjunctive treatment (IMP2 + 2φ MA3) significantly inhibited the growth of KP20 in vitro compared to the two-phage treatment alone (*p* = 0.04). At MOI 0.001, the two-phage cocktail combined with imipenem had significantly lysed the bacterial cell compared to the two-phage cocktail. This statistical difference might be related to the beneficial effect of bacteriophage treatment in adjunctive therapy. Our finding contradicted the study of Pacios et al., who indicated the inability of phage-imipenem combination to kill imipenem-resistant isolate harbouring OXA-245 b-lactamase.^38^ This divergence might be related to the use of a single lytic phage in adjunctive treatment instead of phage cocktail. Furthermore, an antibacterial effect was also observed between the phage cocktails in combination with the most effective antibiotic (tigecycline) without significant difference (*p* = 0.99). A number of studies have reported the positive effect of bacteriophages in combination with antibiotics.^39^ This synergistic activity between phage cocktail in combination with imipenem might have been due to the sensitivity of the bacterial strains to the select antibiotic after bacteriophage action. Indeed, it was reported in a previous study that phage-resistant bacterial strains are more susceptible to antibiotics and the rate of their growth is slower in comparison with wild bacterial strains.^40^ Our study also demonstrated the efficacy of phage cocktail in the absence of phage receptor analysis in formulating phage cocktails. Interestingly, this study pointed out the repurposing of imipenem using phage cocktail therapy, and further encourages the repurposing of the efficacy of Food and Drug Administration-approved antibiotics for acceptance of phage therapy worldwide, especially in Africa.

### Limitations

The limitation of this study was the absence of genomics, which could enable us to further conclude on the taxonomic classification of the bacteriophages. The conclusions were made based on a single carbapenem-resistant strain, which was also a limiting factor in our study due to the high host specificity of phages, and the diverse *K. pneumoniae* strain types and resistance mechanisms which may affect the utility of the adjunctive therapy. The potential impact of phages on the normal microbiota was not considered in the current study, especially given that the phage cocktails might have activity against *K. pneumoniae* strains other than those that are carbapenem-resistant, and *E. coli* strains.

### Conclusion

This current study revealed the presence of lytic tailed *Klebsiella* phages belonging to the family *Myoviridae, Siphoviridae and Podoviridae* in Nairobi sewage systems with relatively short latent periods and optimal burst sizes, indicating their therapeutic potential in composing phage cocktails and synergistic interaction in combination with non-sensitive antibiotic (imipenem) against carbapenem-resistant *K. pneumoniae* clinical isolate in vitro.
