# Corrigendum: Formulation of phage cocktails and evaluation of their interaction with antibiotics in inhibiting carbapenemase-producing *Klebsiella pneumoniae* in vitro in Kenya

**DOI:** 10.4102/ajlm.v12i1.2028

**Published:** 2023-05-09

**Authors:** Noutin F. Michodigni, Atunga Nyachieo, Juliah K. Akhwale, Gabriel Magoma, Abdoul-Salam Ouédraogo, Andrew N. Kimang’a

**Affiliations:** 1 Department of Molecular Biology and Biotechnology, Pan African University Institute for Basic Sciences Technology and Innovation (PAUSTI), Nairobi, Kenya; 2 Department of Reproductive Health and Biology, Institute of Primate Research (IPR), Nairobi, Kenya; 3 Department of Zoology, School of Biological Sciences, Jomo Kenyatta University of Agriculture and Technology (JKUAT), Nairobi, Kenya; 4 Department of Biochemistry, College of Health Sciences, Jomo Kenyatta University of Agriculture and Technology (JKUAT), Nairobi, Kenya; 5 Department of Medical Microbiology Laboratories, Souro-Sanou Teaching Hospital, Bobo-Dioulasso, Burkina Faso; 6 Department of Medical Microbiology, College of Health Sciences, Jomo Kenyatta University of Agriculture and Technology (JKUAT), Nairobi, Kenya

In the published article, Michodigni NF, Nyachieo A, Akhwale JK, Magoma G, Ouédraogo A-S, Kimang’a AN. Formulation of phage cocktails and evaluation of their interaction with antibiotics in inhibiting carbapenemase-producing *Klebsiella pneumoniae* in vitro in Kenya. Afr J Lab Med. 2022;11(1), a1803. https://doi.org/10.4102/ajlm.v11i1.1803, on page 1 the following paragraph is updated as it was incorrectly formulated:

**The original incorrect wording:**

The precipitated bacteriophages were members of *Myoviridae, Siphoviridae and Podoviridae*.

**The revised and updated wording:**

The precipitated bacteriophages were members of *Myoviridae* and *Podoviridae*.

In addition, on page 7 the following paragraph is updated as it was incorrectly formulated:

**The original incorrect wording:**

This current study revealed the presence of lytic tailed Klebsiella phages belonging to the family *Myoviridae, Siphoviridae and Podoviridae* in Nairobi sewage systems with relatively short latent periods and optimal burst sizes, indicating their therapeutic potential in composing phage cocktails and synergistic interaction in combination with non-sensitive antibiotic (imipenem) against carbapenem-resistant *K. pneumoniae* clinical isolate in vitro.

**The revised and updated wording:**

This current study revealed the presence of lytic tailed *Klebsiella* phages belonging to the family *Myoviridae* and *Podoviridae* in Nairobi sewage systems with relatively short latent periods and optimal burst sizes, indicating their therapeutic potential in composing phage cocktails and synergistic interaction in combination with non-sensitive antibiotic (imipenem) against carbapenem-resistant *K. pneumoniae* clinical isolate in vitro.

The authors apologise for these errors. The corrections do not change the study’s findings of significance or overall interpretation of the study’s results or the scientific conclusions of the article in any way.

